# Epiploic Appendagitis: An Important Differential Diagnosis

**DOI:** 10.7759/cureus.67750

**Published:** 2024-08-25

**Authors:** Nghi Khuat, Sanjana Konda, Latha Ganti

**Affiliations:** 1 Biomedical Sciences, University of Central Florida, Orlando, USA; 2 Public Health, Brown University, Providence, USA; 3 Emergency Medicine and Neurology, University of Central Florida, Orlando, USA; 4 Research, Orlando College of Osteopathic Medicine, Winter Garden, USA; 5 Medical Science, The Warren Alpert Medical School of Brown University, Providence, USA

**Keywords:** internal inflammation, emergency medicine, acute ischemic necrosis, rare cause of acute abdominal pain, epiploic appendagitis

## Abstract

Epiploic appendagitis is a rare, often misdiagnosed condition that causes acute abdominal pain. The symptoms, such as localized pain that worsens with coughing and stretching, mimic other conditions like appendicitis and diverticulitis. Diagnosis can be made using computed tomography (CT) scans, which show characteristic signs, such as a 2-3 cm fat-density ring, colon wall thickening, and nearby fluid or inflammation. The condition usually resolves naturally or can be treated with nonsteroidal anti-inflammatory drugs (NSAIDs). In this report, a case of a 37-year-old man diagnosed with epiploic appendagitis in the Emergency Department (ED) is presented.

## Introduction

Epiploic appendagitis is an uncommon medical condition that is characterized by inflammation of the epiploic appendages, resulting in abdominal pain [1]. The epiploic appendages are small, fat-filled pouches that protrude from the serosal surface of the colon, measuring 1-2 cm in thickness and 0.5-5 cm in length [2]. They are predominantly located along the left side of the colon [1].

While their exact physiological function remains uncertain, several theories have been proposed. One theory suggests they act as minor blood reservoirs, providing cushioning, immune functions, protection against inflammation, and aiding fluid absorption [2]. An alternative theory is that the appendages serve as fat storage depots, enabling the utilization of fat during periods of low caloric intake [3].

The primary cause of epiploic appendagitis is torsion or twisting of the appendage, leading to vascular compromise. This obstruction of blood flow results in ischemia, necrosis of the appendage tissue, and subsequent inflammation and thrombosis [4]. The most common sites for epiploic appendagitis are the rectosigmoid region (57%) and the ileocecal region (26%), though it can also occur less frequently in the ascending (9%), transverse (6%), and descending (2%) colon [5]. Therefore, patients usually present with acute, severe pain in the lower left abdominal quadrant [6]. This pain can be aggravated by movements that stretch the abdominal wall, such as coughing [6]. Epiploic appendagitis can be diagnosed through cross-sectional imaging techniques like computed tomography (CT) or abdominal ultrasonography [7].

While epiploic appendagitis can occur in individuals of any age and gender, it predominantly affects obese middle-aged and older adult males [1]. Precise incidence rates are unknown, but estimates suggest 2-7% of suspected acute diverticulitis cases and 0.3-1% of suspected acute appendicitis cases are actually epiploic appendagitis [7]. Misdiagnosis is common due to the commonality of symptoms with other acute abdominal conditions like diverticulitis, appendicitis, and cholecystitis [8].

A key distinguishing factor is that epiploic appendagitis does not require surgical intervention, unlike appendicitis. Therefore, increasing awareness and accurate diagnostic skills for this condition are crucial to avoid unnecessary surgical procedures that could stem from misdiagnosis based on imaging studies.

## Case presentation

A 37-year-old male presented to the emergency department (ED) with a primary complaint of abdominal pain. Although there was right lower quadrant tenderness, the patient did not exhibit any symptoms of fever, chills, diarrhea, vomiting, or changes in bowel habits. Moreover, the patient had no history of previous appendicitis or diverticulitis. Vital signs in the emergency department were 36.2^o^C, respiratory rate 19 breaths per minute, pulse 87 beats per minute, blood pressure 137/90 mmHg, with room air oxygenation 97%.

During the physical examination, the patient did not appear to be in acute distress and his vital signs were stable. Laboratory analyses, including white blood cell count, were unremarkable. He was then sent to the radiology department for a contrast CT scan of the abdomen and pelvis.

**Figure 1:**
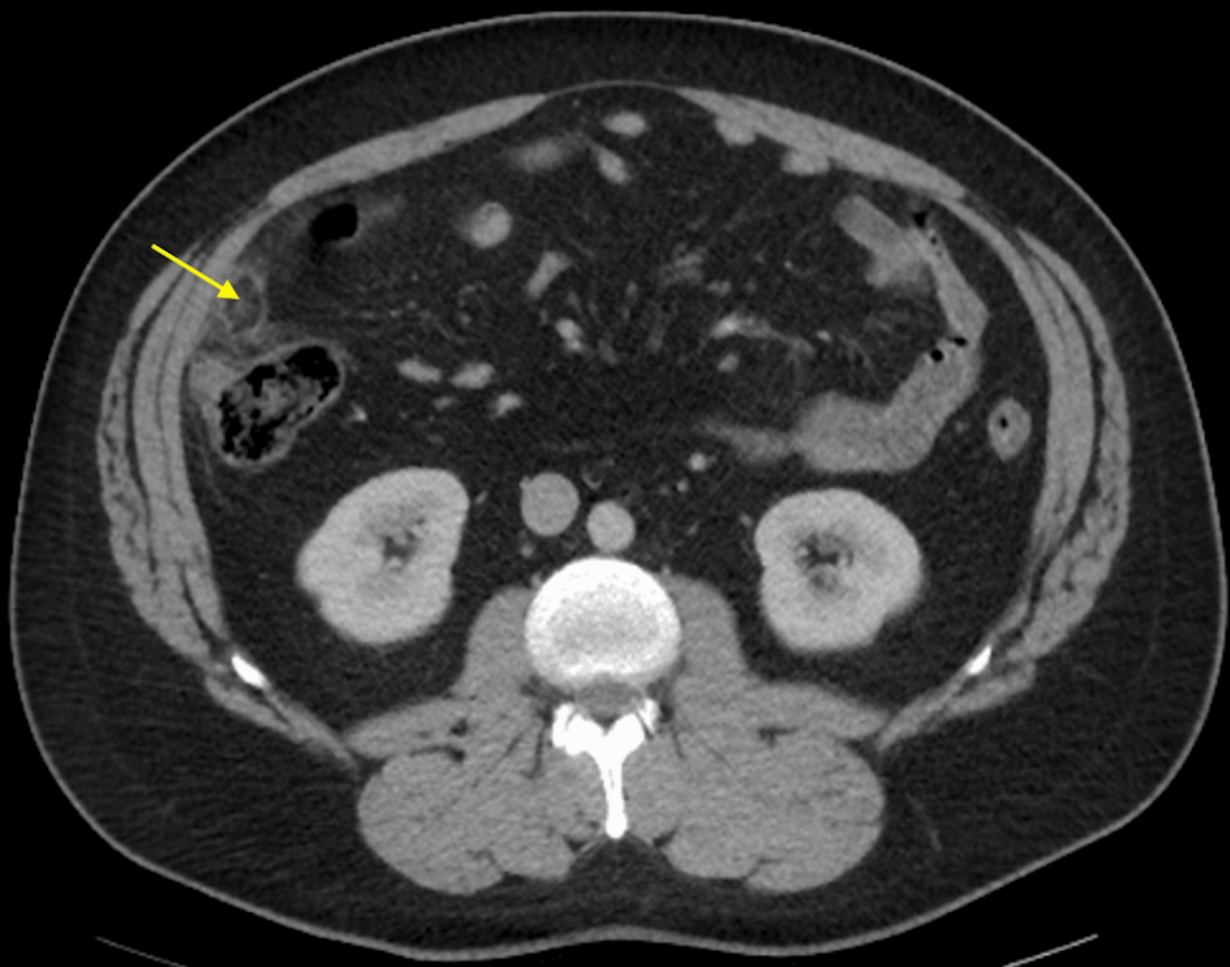
Computed Tomography (CT) scan with highlighted epiploic appendagitis of the ascending colon

The CT scan revealed normal solid abdominal viscera in the upper abdomen and unremarkable osseous structures. The appendix, measuring 5 mm in diameter, showed no apparent signs of appendicitis. There was however inflammation with a central fat density in the ascending colon (Figure 1).

These imaging findings led to the diagnosis of epiploic appendagitis of the ascending colon. The patient was given intravenous ketorolac for analgesia and discharged home with primary care follow-up.

## Discussion

This case highlights a relatively less-known condition - epiploic appendagitis. It highlights the importance of accurate diagnosis for ensuring the most effective treatment while also emphasizing the vital role of advanced imaging techniques, such as CT scans, in differentiating between conditions with similar symptoms [7]. Therefore, it is important to note the critical role of CT imaging in accurately identifying epiploic appendagitis.

Typically, CT imaging depicts epiploic appendagitis as a 2-3 cm, oval-shaped, fat density, parabolic mass with a thickened peritoneal lining, periappendiceal fat stranding, and a 1-3 mm hyperattenuating ring sign [5]. Alternatively, epiploic appendagitis could present as a high-attenuated central dot within the inflamed appendage, which corresponds to a thrombosed draining appendageal vein [2]. However, without any sign of inflammation, epiploic appendages usually are not visible on the CT scan, unless they are surrounded by intraperitoneal fluid [4]. While epiploic appendagitis commonly occurs in the anterior section of the sigmoid or descending colon, this case demonstrates that it can also occur in the ascending colon, which accounts for only 9% of all epiploic appendagitis cases [5].

**Figure 2:**
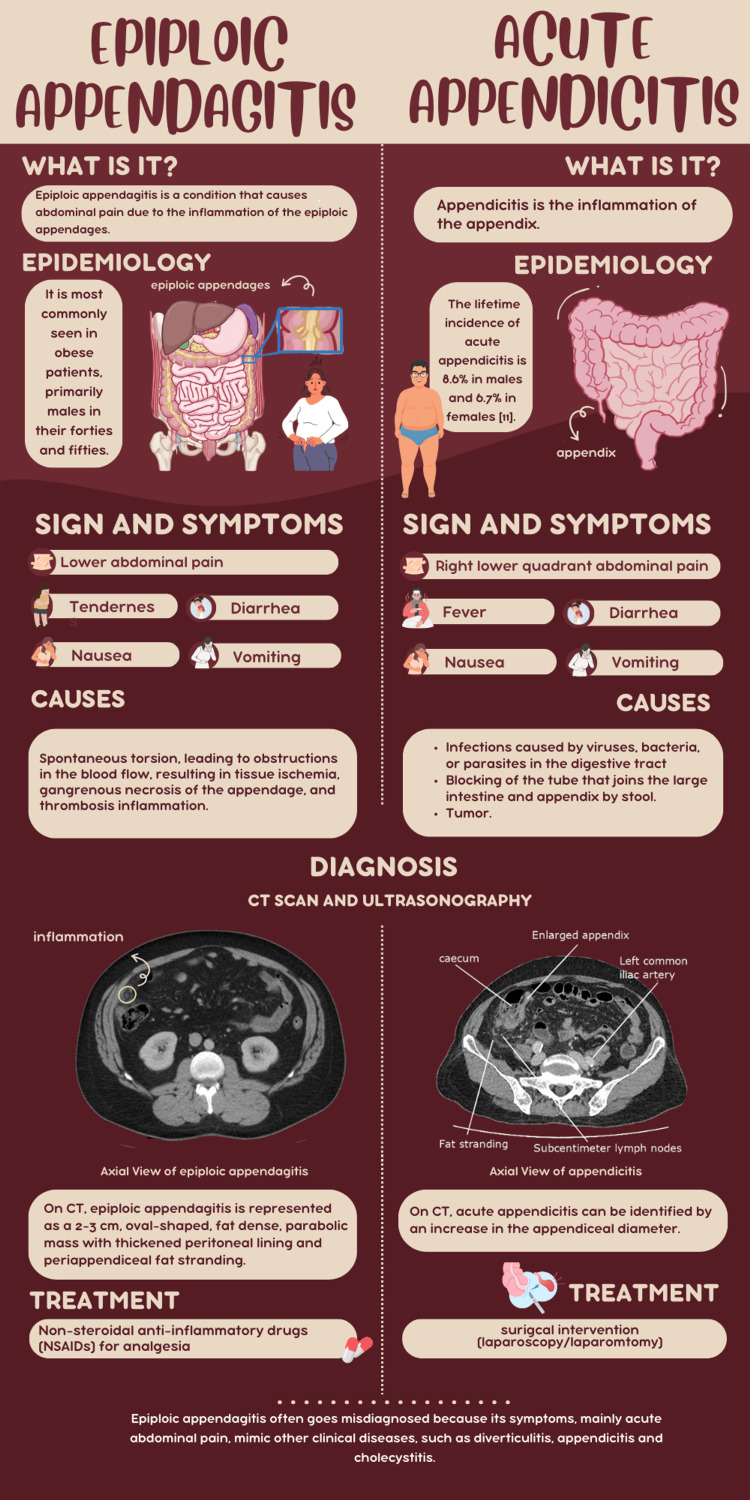
Infographic comparing epiploic appendagitis and acute appendicitis. Designed by Nghi Khuat on Canva.com

The torsion of the epiploic appendages can lead to ischemia and infarction, which is what results in abdominal pain [4]. However, depending on the location of the appendages, this abdominal pain could be easily misdiagnosed as appendicitis. For example, acute epiploic appendagitis of the appendix, which is inflammation of the epiploic appendages near the vermiform appendix, is commonly misinterpreted as acute appendicitis (Figure 2) [9].

Diverticulitis is another condition that shares the symptoms of abdominal pain. Despite the similar symptoms, CT imaging can be used to distinguish between these conditions. While epiploic appendagitis presents with a thickened colon layer, diverticulitis does not show signs of this thickening of the colon [10].

In contrast, patients with epiploic appendagitis typically recover with analgesic medications, such as nonsteroidal anti-inflammatory drugs (NSAIDs) [3]. Therefore, misdiagnosing epiploic appendagitis as another condition often leads to unnecessary hospitalizations, surgical interventions, and antibiotic therapy, which can all be prevented with accurate diagnosis and management [8].

## Conclusions

Unlike other abdominal diseases, such as appendicitis or cholecystitis, which require proper operative intervention, epiploic appendagitis can be treated with analgesic medications. Therefore, it is important to recognize epiploic appendagitis in the differential of right lower quadrant pain, to prevent unnecessary surgical intervention and misdiagnosis. In the case presented, the patient was able to be treated with intravenous ketorolac, a nonsteroidal anti-inflammatory drug, and be discharged from the emergency department. Epiploic appendagitis is a benign self-limited condition that does not require antibiotics or surgery and is managed conservatively with non-opiate analgesia.
